# Analysis of Fatty Acid Composition in Sprouted Grains

**DOI:** 10.3390/foods12091853

**Published:** 2023-04-29

**Authors:** Boris Nemzer, Fadwa Al-Taher

**Affiliations:** 1 Research & Development, VDF/FutureCeuticals, Inc., Momence, IL 60954, USA; bnemzer@futureceuticals.com; 2 Department of Food Science and Human Nutrition, University of Illinois at Urbana-Champaign, Urbana, IL 61801, USA

**Keywords:** sprouted grains, germination, lipids, essential fatty acids, omega-3, omega-6, stability

## Abstract

A whole-grain diet is associated with the prevention of metabolic syndromes, including obesity, diabetes, and cardiovascular diseases. Sprouting improves the nutritional profile and bioactive properties of grains, which are important for use as raw ingredients in the food industry. The aim of this review was to examine the lipid and fatty acid composition of germinated grains. The methods discussed include germination and analytical procedures for determining fat and fatty acid contents of grains. The effects of sprouting on the fat content and storage stability of grains were also assessed. Lipid levels ranged from 1.43% to 6.66% in the sprouted grains. The individual fatty acid content of grains changed depending on the germination conditions (17–37 °C, 1–9 days). Limited findings showed that sprouting grains at higher temperatures (20–25 °C) and longer times generated a healthy balance of omega-6 and omega-3 fatty acids, which is beneficial to humans. Future studies are needed to determine the optimum incubation and germination periods specific to each grain to improve the omega-6/omega-3 ratio. Free fatty acids were produced more slowly and levels of oxidation products were lower in sprouted grains than in the raw ingredients when stored for a year. Additional studies are required to investigate the oxidative stability and shelf life of sprouted grains.

## 1. Introduction

Whole-grain diets present significant health benefits, including decreased weight gain and reduced risks of diabetes and cardiovascular diseases [1]. The outer layer of the whole grain, the bran, consists primarily of non-digestible carbohydrates and protects the inner layers from external environmental pressures. The inner layers are the endosperm and germ, which are rich sources of carbohydrates, proteins, soluble fibers, resistant starch, vitamins, minerals, antioxidants, lipids, and other phytochemicals [1]. Efforts are being made to enhance the nutritional quality and functional properties of grains through germination to improve consumers’ health [2]. In 2008, the American Association of Cereal Chemists (AACC) defined sprouted grains as “malted or sprouted grains containing all of the original bran, germ and endosperm shall be considered whole grains as long as sprout growth does not exceed kernel length, and nutrient values are not diminished” [3,4]. 

During seed sprouting, metabolic changes occur whereby hydrolytic enzymes are synthesized, which break down complex molecules to form simpler ones, making nutrients available for plant growth and development. Sprouting enhances the nutrient profile and bioactive properties of grains. Sprouted grains contain higher amounts of vital nutrients, such as vitamins, proteins, essential minerals, trace elements, and antioxidants, than the raw ingredients and have better bioavailability of some of these nutrients than non-germinated grains [4,5,6,7]. They also contain a more beneficial amino acid composition and higher polyunsaturated fatty acid content. Trypsin inhibitor activity and levels of antinutritive compounds, hemagglutinins, tannins, pentosans, and phytic acid are decreased in sprouted grains [1,7]. Any cereal and leguminous seed consumed, such as wheat, rice, maize, sorghum, barley, millet, rye, oats, mug beans, soybeans, and black beans, can be used for sprouting [6]. Sprouting is essential for the industrial use of raw ingredients as functional foods. 

The use of sprouted grains as ingredients in food products is becoming prevalent and is an emerging trend among healthy foods in the marketplace [4,5,6]. Sprouts may be eaten whole, dried, or prepared as flour. The rate of food products containing sprouted grains has increased considerably since 2006. However, the largest change occurred from 2012 to 2016, with an increase of 26%. These products included snacks (22%), flour (19%), and bakery products (15%) [8]. Non-alcoholic beverages, such as kombucha, kefir, and vegetable and fruit juices or infusions, have become a trend in cuisine. Nutritional and functional beverages generated from lactic acid fermentation of a mixture of sprouted grains and flours are possible food products on the market [4]. The food industry has marketed sprouted grain and flour products in the U.S. and Europe ranging from baked goods to pasta, breakfast cereals, snacks, and beverages [9]. New products include bread, biscuits/crackers, baking ingredients and mixes, sweet biscuits/cookies, cakes, pastries, and sweet goods [8]. 

Cereal grains contain several monounsaturated and polyunsaturated fatty acids, which lower cholesterol and triacylglycerol concentrations while improving insulin resistance [10,11,12]. However, saturated fatty acids increase the risks of cancer and cardiovascular and immune system diseases [13]. The major fatty acids in grains are palmitic, oleic, and linoleic acids, with minor levels of alpha-linoleic and arachidic acids and trace levels of gadoleic and behenic acids [11,12,14]. A wholesome diet should contain omega-3 and omega-6 fatty acids. Balancing omega-6 and omega-3 fatty acids is imperative to reduce health risks. The recommended ratio of omega-6/omega-3 fatty acids is 2:1, which is important for decreasing the risk of coronary heart diseases [15]. 

As sprouted grain flours are used to produce cereal-based products such as bread, cookies, cakes, and muffins and there is a considerable amount of data on the nutrient and phytochemical composition of sprouted grains, it is important to evaluate the lipid and fatty acid contents of germinated grains since these data are lacking. The aim of this comprehensive review was to determine the grain with the most abundant beneficial fatty acids and the lowest omega-6/omega-3 fatty acid ratio to reduce the risks of metabolic syndromes. 

## 2. Sprouted Grains and Health Benefits

The nutritional composition of a germinated grain depends on the grain type and germination conditions, and germinated grains may have different health effects when consumed compared to non-germinated grains. Germination may considerably boost the nutritional profile and levels of bioactive compounds in grains and enhance tastiness [16].

Sprouted grains can be eaten whole, dried, or ground into flour. Quinoa (a pseudocereal) and wheat are the most commonly consumed sprouted grains. Sprouted buckwheat, barley, and millet have also been used but are less popular [9]. Germinated wheat, quinoa, oats, sorghum, millet, barley, rye, and brown rice are used to make bread. Yogurt is also prepared from sprouted wheat, and cookies are made from sprouted maize and brown rice [6]. 

Consumption of whole wheat decreases the incidences of colon cancer, diabetes mellitus, and cardiovascular diseases. However, it also contains antinutrients that can inhibit the absorption of nutrients and digestive enzymes. Germination was found to decrease antinutrient levels and improve the bioavailability of nutrients in wheat [17]. Germinated wheat can be used as sprouts and flour in breakfast items, salads, soups, casseroles, pasta, and baked goods [17]. Sprouted grains can replace grains with low nutritional value, such as wheat flour, during baking [7]. 

Sprouted grains have high bioavailability, excellent sensory characteristics, an extended shelf life, and low antinutrient content. Consumption of sprouted grains increases metabolism, improves immunity, and enhances the nutritional content of baked goods. Sprouted grains are a good source of proteins, dietary fiber, folate, vitamins, and minerals [6]. They also contain higher amounts of polyunsaturated fatty acids than the raw ingredients [18].

Omega-6 and omega-3 polyunsaturated fatty acids are essential fatty acids that cannot be synthesized by humans but must be obtained from the diet. Omega-6 fatty acids are characterized by linoleic acid (LA) (C18:2ω-6), and omega-3 fatty acids are depicted by alpha-linolenic acid (ALA) (C18:3ω-3). Both essential fatty acids are metabolized to longer chains: LA to arachidonic acid (AA) (C20:4ω-6) and ALA to eicosapentaenoic acid (EPA) (C20:5ω-3) and docosahexaenoic acid (DHA) (C22:6ω-3) [19]. Polyunsaturated fatty acids have several beneficial effects on health, such as improving the lipid profile, decreasing the occurrence of type 2 diabetes, and reducing the incidence of cardiovascular diseases [20].

An important preventive measure against diseases is the intake ratio of omega-6 to omega-3 fatty acids. Historically, the ratio was approximately 1:1; however, owing to Western diets, it has reached an unhealthy level of 20:1, with large amounts of omega-6 fatty acids and a very small omega-3 fatty acid content [19]. High amounts of omega-6 fatty acids cause an elevated omega-6/omega-3 ratio, which promotes the development of many illnesses, such as cardiovascular diseases, cancers, and autoimmune diseases [19]. This is also linked to weight gain; however, high omega-3 fatty acid intake results in homeostasis and weight loss. High intake of omega-6 fatty acids also increases insulin resistance. Lowering the omega-6/omega-3 ratio prevents obesity, and an omega-6/omega-3 ratio of 1–2/1 is suggested to inhibit obesity [19]. Consumption of omega-3 fatty acids has been associated with beneficial health effects, such as a lower incidence of cardiovascular diseases, whereas ingesting large amounts of omega-6 fatty acids may lead to thrombosis and inflammation, contributing to atherosclerosis, obesity, and diabetes [21,22,23]. Therefore, it is important to reduce omega-6 fatty acid consumption and increase omega-3 fatty acid intake [19].

## 3. Germination

### 3.1. Germination Process

Several changes occur during seed sprouting. Sprouting revives seed uptake, causing the catabolism and degradation of macronutrients and antinutritive compounds and the biogenesis of secondary metabolites with possible health advantages [24]. Germination can increase the nutritional value and functional compound content of grains as well as their flavor, digestibility, and bioavailability [2]. 

The grain is soaked (steeped) in water for 8–24 h until it reaches an optimum moisture content (imbibition) and the cell membrane and media become completely hydrated [5,25]. Hormones are then released to stimulate the production of the hydrolytic enzymes amylase, protease, and lipase, which break down proteins, starches, and lipids, supplying the developing embryo with energy [5,7,26,27]. The grain is then drained and transferred to a germination vessel, where nutrients are used for sprouting. In the final step, the grain is moved to a germination vessel or kiln and dried at varying times and temperatures specific to each grain type until the appropriate moisture content is achieved [5,6,26]. Germination causes biochemical, nutritional, and sensory changes. The primary and secondary metabolites generated have different health effects compared to those of non-germinated seeds [28]. 

Germinated grains are easier to digest than non-germinated grains. During germination, several enzymes are released that break down complex molecules into simple compounds. Proteins are degraded, causing an increase in the levels of peptides and amino acids, and carbohydrates are converted to oligosaccharides and simple sugars [6,7,8]. However, lengthy and uncontrolled germination can result in the increased generation of hydrolytic enzymes, causing the deterioration of bread flour [7]. 

Food products made from sprouted grains satisfy consumer demand for nutrition, health, and taste. A beneficial sensory characteristic of sprouted grains is flavor. The conversion of starch into simple sugars and oligosaccharides adds natural sweetness to products containing sprouted grains, allowing food manufacturers to add lower amounts of sugar to these products [8]. More than 30 volatile flavor compounds were found in heat-treated rye malt extracts, including pyrazines, pyrazoles, pyranones, pyridines, pyrimidines, furans, furanones, phenols, esters, aldehydes, ketones, and alcohols. The unique flavor of sprouted grains distinguishes them from their non-sprouted counterparts [8]. 

In an expert sensory panel study, non-sprouted multigrain bread was described as possessing a “malted, yeasty, nutty aroma with herbal notes,” while sprouted multigrain bread was portrayed as having “naturally sweet, malted, fermented, fruity, and roasted notes [8].” This study indicated that consumers preferred bread made with flavorful sprouted grains to bread made with non-sprouted grains.

### 3.2. Germination Conditions 

Finnie et al. [27] maintained germinated seeds in an aerobic environment with steady and continuous airflow and temperature for uniform germination. Cardone et al. [29] noted that controlling the germination process was essential for formulating flour for the bread-making process because uncontrolled germination can lead to large accumulations of enzymes that can deteriorate bread quality. It is important to regulate moisture, temperature, and germination duration the sprouting of cereal grains [6,30]. Controlled germination can increase the nutritional value of sprouted grains to obtain better quality raw materials or ingredients for food processing [30]. Each grain type has varying temperatures and times specific to its growth [7,27]. The conditions depend on factors such as species, genetic variation, variety, seed source, and age. Optimum germination temperatures are between 20 °C and 30 °C, with a few cases of temperatures above 30 °C, such as maize and rice at about 35 °C. Cereal grains can germinate with or without light [6]. Long sprouting times (3 to 5 days) and high processing temperatures (25 °C to 35 °C) increase the release of plant bioactive compounds [9].

## 4. Analytical Methods for Determination of Fats in Grains

### 4.1. Crude Fat Determination

Official methods for fat analysis in foods have been evaluated and approved by authorized organizations that develop procedures for routine analysis in regulatory, contractual, and other laboratories. Crude fat can be extracted using published AOAC or AOCS methods. AOAC Official Method 948.22 [31] is a solvent extraction procedure for determining the crude and total fat contents of nuts and nut products. Samples are extracted with ether in a Soxhlet-type extractor for 16 h, after which the extracted lipid is dried at 95–100 °C and weighed. Modifications of the Soxhlet solvent extraction procedure include the Randall/Soxtec/diethyl ether (AOAC 2003.05) [32] and Randall/Soxtec/hexane (AOAC 2003.06) [33] extraction–submersion methods, which provide a shorter extraction time because the sample is submerged in boiling solvent. AOCS Official Method Am 5-04 [34] was approved for rapid determination of the total fat content of oil seeds, meats, feeds, and foods using an automated or semi-automated extraction system. Substances extracted using petroleum ether are predominantly triacylglycerides, with small amounts of other lipids and minor components. 

An alternative to the solvent extraction method is a two-step hydrolysis process in which the sample is pretreated with acids, alkaline reagents, or enzymes to break down the matrix before extraction with a solvent. Hydrolytic treatment disrupts the lipid–carbohydrate bonds, proteins, polysaccharides, and plant cell walls. AOAC Official Method 983.23 [35] describes a hydrolytic method for determining the fat content of foods. 

### 4.2. Fatty Acid Determination 

Interest has grown in determining the content and composition of fatty acids in foods and food ingredients, prompting the development and validation of analytical methods for quantifying fatty acids using gas chromatography with flame ionization (GC–FID) or gas chromatography–mass spectrometry (GC–MS) [36,37]. AOAC Official Method 996.01 [38] was approved for determination of the fat content of cereal products containing 0.5–13% total fat using a gas chromatographic method. The sample is heated while being shaken in ethanol and 8 M HCl at 80 °C for 40 min and then cooled. Subsequently, it is transferred with ethanol to a Mojonnier fat extraction flask for liquid–liquid extraction with ethyl and petroleum ether, followed by evaporation with nitrogen. The extract is saponified and methylated by the addition of sodium hydroxide (0.5 M) in a methanol solution and then esterified with 14% boron trifluoride in methanol. Fatty acids are derivatized to fatty acid methyl esters (FAMEs) and quantified using GC–FID or GC–MS. The total fat content is determined by the summation of individual fatty acids [36,37,39,40]. Conversion factors are used to express the analyzed FAMEs as free fatty acid equivalents. It is difficult to analyze fatty acids in their free forms, as they are highly polar and will form hydrogen bonds, causing problems with adsorption [41]. AOAC Official Method 996.06 [42] demonstrates an interlaboratory collaborative study for determining the fat content (total, saturated, and unsaturated) in food matrices, including wheat-based cereal products, with a total fat content of 1.5–46%.

## 5. Effects of Sprouting on the Fat Content of Grains

### 5.1. Lipase Activity

Lipids are abundant in the embryo, scutellum, and aleurone of whole grains as oil (triacylglycerols) [28,43,44]. Lipid catabolism provides energy and carbon sources for biochemical and physicochemical changes during seed growth. 

During germination, lipases release esterified fatty acids from triglycerides [43,44]. As lipases are activated, triacylglycerols are hydrolyzed into free fatty acids, increasing the saturated/unsaturated fatty acid ratio [28]. Free fatty acids are then degraded through β-oxidation and the glyoxylate cycle and converted to sugars [44]. This may result in the development of off-flavors [45].

Long sprouting times enhance undesirable flavors and odors due to increased lipase and lipoxygenase levels leading to aldehydes, free phenolic compounds, heterocyclic substances, and dimethyl sulfide [46,47,48]. 

Lipase acts as soon as the grain structure breaks down and comes into contact with its substrates [45]. Kubicka et al. [44] noted that lipase activity increased 1.2- to 2.3-fold during the sprouting of cereal grains because of the synthesis of lipase in the outermost part of the endosperm. However, oats are unique and contain high lipase activity in their grains that is either maintained or reduced during sprouting [49]. Aparicio-Garcia et al. [45] demonstrated that lipase activity was reduced by 17% in sprouted oats compared to that in the control. It was also observed that the degradation of oil from the oat embryo occurred earlier than that of oil reserves in the scutellum [50]. One to two days after imbibition, triglyceride mobilization begins in the endosperm of oats, with the accumulation of free fatty acids [50]. Aparicio-Garcia et al. [51] showed that lipase activity increased in a sprouted hulled oat variety but was reduced in a dehulled oat variety. Heiniö et al. [47] observed that hulled oats had a lower lipid content than hull-less oats. A greater amount of triglycerides (>75%) was hydrolyzed in hulled oats steeped in water for 15 h than in dehulled oats (15%). The lipase activity was higher in oat grains than in sprouted oats. Hosseini et al. [52] found greater amounts of free fatty acids in hulled than in dehulled sprouted oats, suggesting an important role in the increased lipid activity in oat hulls.

### 5.2. Lipid Content of Sprouted Grains

Lipid amounts in non-germinated and germinated grains were evaluated and compared using data from various studies. The lipid content was higher in sprouted oats after germination at 25 °C/60% relative humidity [53]. The fat content decreased by 8–15% owing to lipase activity in sprouted millet (3 days, 20–23 °C) [54]. No change was observed in the fat content of sprouted wheat compared to that of seeds when germinated for 3 days at 20 °C [55]. Farooqui et al. [56] reported that the fat content decreased from 2.75% in non-germinated barley to 2.10% when barley was sprouted at 25 °C for 72 h. However, Ortiz et al. [57] observed the amount of fat to be 50.2% greater in the sprouted barley than in the raw barley grain when grown under light for 6 days at 20 °C. The total fat content increased from 1.62% in buckwheat grains to 2.42% in sprouted buckwheat when germinated in the dark for 3 days at 30 °C [58]. Conversely, Rico et al. [2] noticed that the fat content of sprouted barley decreased considerably compared to that of the control when grown in the dark at 12.1–19.9 °C and for 1.6–6.19 days. Jiménez et al. [59] did not notice a significant change in the lipid content when sprouting quinoa for 24 h at 22–24 °C and amaranth for 48 h in the dark.

Other studies have demonstrated that higher amounts of lipids are broken down as the sprouting temperature increases. The total lipid content was reduced by 18–28% in the following: sprouted millet (2 days; 32 °C) [60], sprouted wheat (2 days, 30 °C) [61], and sprouted brown rice (1–5 days, 25 to 30 °C) [62,63]. 

Pîrvulescu et al. [64] determined that the highest lipid content from barley, wheat, and oats was found in the germinated Capo wheat genotype (11.12 g/100 g flour). The lipid content of the flours from the non-germinated grains was lower than that of the germinated grains. 

In general, the fat content was lower in sprouted quinoa (0.1–3.8%) [65,66] than in its grain (4.0–7.6%) [67]. The fat content of sprouted grains generally decreases because of the use of lipids as an energy source during germination [68] or increased lipolytic activity, which converts fats into fatty acids and glycerol [69,70,71]. Triglycerides were the most abundant lipids in non-germinated and germinated oil seeds. Changes during sprouting depend on the crop type, genotype, and germination conditions [59].

Table 1 compares the % lipid content of non-germinated and germinated grains derived from previous studies.

### 5.3. Fatty Acids in Sprouted Grains

Table 2 summarizes the results of studies that investigated the effect of germination on the fatty acid content of grains. Individual fatty acid results are presented as percentages of the total fatty acids. Individual fatty acids with concentrations less than 1% of total fatty acids are not listed in this table.

#### 5.3.1. Wheat

Waxy wheat (*Triticum aestivum* L.) has exceptional properties for forming bread textures with soft, thick, and gelatinous breadcrumbs that prevent bread staleness. Wheat contains large amounts of essential fatty acids (linoleic and linolenic acids) that the human body cannot synthesize because humans do not have the enzymes required for their production [61]. 

Hung et al. [61] found that ungerminated and sprouted wheat contain eleven fatty acids representing 98.6–100% of total fatty acids. The major fatty acids in order of abundance are linoleic (C18:2), palmitic (C16:0), oleic (C18:1), linolenic (C18:3), and stearic (C18:0) acids. Trace amounts of other components include myristic (C14:0), palmitoleic (C16:1), arachidonic (C20:0), eicosaenoic (C20:1), eicosatrienoic (C20:3), and eicosapentaenoic (C20:5) acids. Hung et al. [61] observed that the fatty acid composition of wheat remained unchanged during 48 h of germination (Table 2). This study concluded that sprouted waxy wheat can enhance the texture and nutritional attributes of cereal-based products.

Ozturk et al. [74] observed that the sprouting of two varieties of wheat (Demir 2000 and Konya 2002) had a significant effect on the fatty acid composition of 9-day-old wheat seeds. Although trace levels of some saturated fatty acids (C4:0, C10:0, and C12:0) were observed in the seeds, they disappeared from the sprouts. The quantity of omega-3 (18:3n-3) fatty acids increased, whereas the amounts of cis-18:1 and cis-18:2 fatty acids decreased when wheat was germinated (Table 2). Márton et al. [55] compared the fatty acid content of wheat seeds and wheat sprouts after 3 days of germination at 20 °C. The predominant fatty acids in seeds and sprouts were palmitic (C16:0), linoleic (C18:2 cis), and oleic (C18:1) acids. While the amounts of palmitic and linoleic acids increased in sprouted wheat, oleic acid levels decreased (Table 2). 

#### 5.3.2. Buckwheat

Buckwheat (*Fagopyrum esculentum*) is unrelated to wheat, unlike its name suggests. It is not a cereal or a member of the grass family but a pseudocereal because its seeds are used similarly to those of cereals for culinary and nutritional purposes. It is gluten-free [75]. 

Kim et al. [73] assessed the fatty acid composition of buckwheat and its sprouts. Oleic (C18:1cis), linoleic (C18:2), and linolenic (C18:3n-3) acids comprised 36.8%, 38.1%, and 2.7% of the total fatty acid composition of the raw ingredient, respectively. The major saturated fatty acid in buckwheat seeds, palmitic acid (C16:0), comprised approximately 17.7% of the total fatty acid content (Table 2). Small amounts of myristic (C14:0), stearic (C18:0), arachidic (C20:0), and behenic (C22:0) acids were detected. The study found that linoleic acid (C18:2) was the most abundant fatty acid in the sprouts, and its content increased by up to 52.1% in 7 days, with the total amount of unsaturated fatty acids (oleic, linoleic, and linolenic acids) being greater than 83% and higher than that of the saturated fatty acids. However, the amount of saturated fatty acids rapidly decreased; myristic and stearic acids disappeared after 1 day of sprouting, and arachidic and behenic acids disappeared after 3 days of sprouting. The greatest decrease among unsaturated fatty acids was observed for oleic acid; however, the amounts of linoleic, linolenic, and arachidonic acids increased in sprouts as germination progressed [73]. Linoleic, linolenic, and arachidonic acids are essential because they cannot be synthesized in the human body. 

Molska et al. [58] germinated buckwheat seeds in the dark for 3 days at 30 °C. Higher concentrations of unsaturated fats, constituting 78.48% of the total fatty acids in seeds and 83.04% in sprouts, were observed than saturated fatty acids, which constituted 21.2% in seeds and decreased to 16.8% in sprouts. The most abundant fatty acids in buckwheat seeds and sprouts were palmitic, oleic, and linoleic acids. However, the quantity of these fatty acids changed during sprouting. The level of palmitic acid significantly decreased in the controlled sprouts (up to 13.90%), but the amount of linoleic acid increased (up to 42.45%) [58] (Table 2). 

Conversely, Yiming et al. [72] observed a different trend in the fatty acid composition of Tartary buckwheat (*Fagopyrum tataricum* L.) when germinated in the dark at 37 °C for up to 7 days. The amounts of palmitic, oleic, and stearic acids increased, whereas those of linoleic and eicosenoic acids decreased (Table 2). 

#### 5.3.3. Quinoa

Quinoa (*Chenopodium quinoa*) is a herbaceous plant grown for its edible seeds. It is not a grass but a pseudocereal that belongs to the Amaranth family. Quinoa is the only plant with all essential amino acids, trace minerals, vitamins, and no gluten [76]. 

Park and Morita [77] studied the fatty acid composition of quinoa seeds germinated for 3 days and noticed that the levels of some fatty acids either increased or decreased. The amounts of saturated and monounsaturated fatty acids increased, whereas those of polyunsaturated fatty acids decreased, and variation was observed in the omega-6/omega-3 ratio. 

Peiretti et al. [78] studied the fatty acid composition of quinoa from the seed to the morphological vegetative stages from the end of June to the end of September 2010. The fatty acids in the seeds were predominantly palmitic (C16:0), oleic (C18:0), and linoleic (C18:2cis) acids. Quinoa contained the highest amount of alpha-linolenic acid (C18:3n-3; omega-3), at 47% of the total fatty acids, whereas linoleic acid (C18:2 cis, omega-6) constituted 16% of the total fatty acids in the early vegetative state. The sprouts contained high levels of linolenic acid (C18:3n-3) (385–473 g/kg of total fatty acids) and polyunsaturated fatty acids (611–691 g/kg of total fatty acids). The linoleic acid content (146–176 g/kg of total fatty acids) decreased significantly in quinoa until the shooting stage was reached and then increased, whereas the amounts of the other fatty acids, palmitic and oleic acids, did not show significant differences during growth. This showed that quinoa grown in the early and mid-vegetative stages contained higher amounts of alpha-linolenic acid and omega-3 fatty acids than linoleic acid and omega-6 fatty acids. Peiretti et al. [78] showed that the omega-6/omega-3 ratio in quinoa was 0.3. Alvares-Jubete et al. [79] reported an omega-6/omega-3 ratio of approximately 1:6.

Jiménez et al. [59] compared the fatty acid profile between the grains and sprouted quinoa grown between 22 °C and 24 °C in the dark for 24 h (Table 2). The amounts of saturated fatty acids, palmitic and behenic acids, decreased, potentially owing to lipase activity and the breakdown of triglycerides and polar lipids into simple compounds during sprouting. As the levels of saturated fatty acids decreased, those of monounsaturated and polyunsaturated fatty acids increased during germination. The omega-6/omega-3 ratio for quinoa grains was 5:24 and decreased to 4:55 when sprouted.

#### 5.3.4. Barley

Barley (*Hordeum vulgare*) is an ancient cereal from the grass family grown in mild climates worldwide and is traditionally used as animal feed and as a raw material for beer and other beverages in the malting industry [80]. There has been heightened interest in its use as a food ingredient because of its nutritional benefits and superior bioactive properties [81].

Rico et al. [2] germinated two varieties of whole-grain barley flour in the temperature range of 12.1–19.9 °C for 1.6–6.19 days in the dark. The fatty acid composition was evaluated and compared with that of the control. No changes were detected in the amounts of saturated, unsaturated, monounsaturated, and polyunsaturated fatty acids in barley grown at lower temperatures and shorter times compared with those of the raw ingredient. However, after a longer germination period, sprouted barley contained approximately 37% more saturated fatty acids than barley grains, whereas the amounts of polyunsaturated acids did not show significant differences, at 59–63%. Rico et al. [2] concluded that the most suitable condition for the sprouting of barley was 16 °C for 3.53 days.

Oritz et al. [57] noticed an increase in the C18:0 (24%) and C18:3n-3 (49%) contents and a decrease in the C18:1n-9 (6%) content in barley sprouted at 20 °C for 6 days in the light. The concentration of linoleic acid (C18:2n-6), which is predominant in barley, did not vary during barley growth. Palmitic acid (C16:0) was the most abundant saturated fatty acid in the sprouted barley (Table 2). Polyunsaturated fatty acids constituted most of the fatty acid composition in green shoots, the residual structure of sprouted grains plus non-sprouted grains, and root fractions ranged from 56.1% to 61.4%. Lower amounts of monounsaturated (14.3–24.6%) and saturated (15.78–21.96%) fatty acids were detected in these sprout fractions [57].

#### 5.3.5. Oat

Oats (*Avena sativa* L.) are cereal grains prepared from crushed or rolled oats and consumed as porridge, flakes, or breakfast cereal. Oat flour can also be used in baked goods. Oats are effective against celiac disease and are used to treat diabetes and cardiovascular disorders [82]. 

Aparicio-Garcia et al. [45] germinated oats at 18 °C for 96 h in the dark. The most abundant fatty acid found in non-germinated and sprouted oats was C18:2n-6 (linoleic acid), followed by C18:1 (oleic acid) and C18:0 (stearic acid) with similar amounts, and C16:0 (palmitic acid). Omega-3 fatty acids (C18:3n-3, linolenic acid) were present in the smallest amounts in oats. Trace levels of other fatty acids were also present (<1% of the total fatty acids). The fatty acid content remained the same or increased slightly as the oats sprouted. The amounts of polyunsaturated fatty acids were higher in the control and sprouted oats than those of monounsaturated and saturated fatty acids (Table 2).

#### 5.3.6. Summary of Findings on Grains

Table 2 shows that sprouted grains, similar to the raw ingredients, contain higher amounts of polyunsaturated fatty acids (56–65%) than saturated fats (11–37%). The amount of these fatty acids either remains constant when sprouted, decreases, or increases slightly depending on the type of grain and germination conditions, including time and temperature. 

Hung et al. [61] did not observe a change in the omega-6/omega-3 ratio (14.0) in wheat when germinated at 30 °C for 2 days. However, a study of two wheat varieties by Ozturk et al. [74] showed a drastic difference in the omega-6/omega-3 ratio when sprouted at the same temperature of 17 °C for 9 days. The omega-6/omega-3 ratio was calculated to be 14.62 and 13.29 for the Demir 2000 and Konya 2002 wheat controls, respectively, and 3.35 and 3.87 for the two wheat sprout types, respectively. This variation was due to the higher omega-3 fatty acid content when the wheat sprouted. This limited study showed that the fatty acid composition does not depend on the genotype of the grain. The omega-6/omega-3 ratio in wheat sprouts was the lowest among the grains evaluated in this review. Márton et al. [55] observed a decline in the omega-6/omega-3 ratio from 12.8 to 10.92 when wheat was sprouted at 20 °C for 3 days. The highest omega-6/omega-3 ratio was 26.2 in oats, which decreased to 22.6 when sprouted at a low temperature of 18 °C for 4 days. This was due to an increase in the amounts of polyunsaturated acids, with no change in the saturated fatty acid content upon germination. These studies suggest that improving the fatty acid content and omega-6/omega-3 ratio by sprouting grains depends on the germination conditions.

## 6. Storage Stability of Grains

During grain storage, lipids may be exposed to hydrolytic and oxidative rancidity owing to aging or unfavorable environmental conditions. Lipids are unstable and may degrade in grains, thereby decreasing the quality of the finished product. Lipase activity, mainly in the bran and germ, exposes lipids to hydrolytic rancidity [47,83]. Lipase enzymes consist of three main groups that act on ester bonds: (1) triacylglycerol lipases, which hydrolyze the ester bonds on triacylglycerols to form diacylglycerols and free fatty acids; (2) phospholipases, which are divided into A1, A2, C, and D depending on the bond they hydrolyze; and (3) galactolipases, which hydrolyze the ester bonds of galactolipids. However, some lipases work on other types of substrates depending on the conditions and activity of the enzyme. For example, wheat germ lipase catalyzes the de-esterification of triglycerides, such as triacetin, and water-soluble substrates [83].

Oxidative rancidity can occur owing to unfavorable storage conditions, primarily temperature and humidity, with the spread of fungi, enzyme activation, and a decline in the quality of grains and flours [47,83].

Although oats have gained popularity owing to their health-promoting properties, their application in foods is limited. The high lipid content of oats poses a challenge to the oat-processing industry in terms of stability and the prevention of off-flavor formation [48,84]. Heiniö et al. [85] observed that the most rigorous lipid hydrolysis in oats occurred in the first 6 months of storage. Less than 50% of the triacylglycerols in oats and germinated grains were converted into free fatty acids, while the number of polar lipids remained the same. The fatty acid composition did not change significantly throughout the storage period. Heiniö et al. [85] noticed that the lower lipolytic activity of oats resulted in slower free fatty acid formation in sprouted oats than in oat grains during the first 2 months of storage. After that period, the accrual of free fatty acids was greater in the germinated oats than in the grains, and the fatty acid composition of the triacylglycerides remained constant throughout the storage period. The oxidation of lipids, indicated by the degree of unsaturation, did not change in germinated oats but was reduced in oat grains [48]. The concentrations of secondary oxidation products of unsaturated fatty acids, volatile aldehydes, and ketones were lower in germinated oats stored for 12 months than in grains [85]. During storage, the lipolytic activity that affects rancidity was lower in the sprouts than in the ungerminated oats [85]. This indicated that the sensory qualities of germinated oats were more desirable than those of grains. Undesirable sensory attributes, such as rancidity and bitterness, were significantly correlated with the accumulation of free fatty acids and volatile lipid oxidation compounds, such as pentanal, 2-ethylfuran, 1-pentanol, hexanal, 1-hexanol, 2-heptanone, n-butylfuran, heptanal, and pentylfuran. Storage stability and flavor improved with germination [48].

## 7. Conclusions

Consumers are interested in foods with greater bioavailability, exceptional sensory qualities, and extended shelf lives. Germinated grains have the potential to be used as functional foods to decrease the risks associated with metabolic syndromes. Sprouting improves digestion and immunity by maximizing the nutritional value of grains. 

The metabolic functions of fatty acids depend on the imbibition and lipase activity in whole-grain tissues. The content and profile of individual fatty acids change depending on the germination conditions. Higher amounts of polyunsaturated fatty acids than saturated fatty acids are observed in the whole grain, and these amounts increase when the grains are sprouted. It can be concluded from these limited studies that sprouting grains at higher temperatures (20–25 °C) and longer times generate a healthy balance of omega-6 and omega-3 fatty acids that is beneficial to humans. Further studies are required to determine the optimum germination conditions (temperature and time) for each grain type.

During grain sprouting, lipases hydrolyze triacylglycerols and release free fatty acids, increasing the saturated/unsaturated fatty acid ratio. Free fatty acids are then degraded and converted to sugars, which may cause off-flavors. Long sprouting times increase the likelihood of undesirable flavors and odors due to increased lipase activity, resulting in oxidation products. Studies have shown that fatty acids are generated more slowly in sprouted grains than in raw ingredients, and concentrations of oxidation products are lower in germinated grains than in raw ingredients when stored for a year. The lower lipase activity in sprouted grains suggests improved levels of functional and nutritional ingredients. However, there are limited studies on the storage stability of germinated grains. Additional experiments are necessary to investigate the oxidative stability and extended shelf lives of sprouted grains.

## Figures and Tables

**Table 1:** Lipid (%) content of grains.

Grains	Non-Germinated	Germinated	References
Oat	4.41 ± 0.20%	5.55 ± 0.01%	[53]
Wheat	1.92 ± 0.66%	1.43 ± 0.26%	[68]
	1.7%	1.7%	[55]
	2.39 ± 0.44%	2.25 ± 0.29%	[17]
Maize	4.36 ± 0.36%	4.28 ± 0.67%	[68]
Millet	4.8 ± 0.70%	3.1 ± 0.10%	[54]
	5.4 ± 0.20%	4.6 ± 0.70%	[54]
Barley	2.45%	3.68%	[57]
	2.75%	2.10%	[56]
Buckwheat	1.62%	2.42%	[58]
Quinoa	7.48%	6.52%	[59]
Amaranth	7.00%	6.66%	[59]

**Table 2:** Effect of germination on individual fatty acid content (% of total fatty acids).

Grains	Fatty Acid	Germination Conditions	Results before Sprouting	Results after Sprouting	References
Quinoa	C16:0 Palmitic acid	22–24 °C/24 h	8.84%	7.08%	[59]
	C18:1cis Oleic acid	22–24 °C/24 h	20.88%	23.37%	[59]
	C18:2cis Linoleic acid (Omega-6)	22–24 °C/24 h	50.55%	52.32%	[59]
	C18:3n-3 Linolenic acid (Omega-3)	22–24 °C/24 h	9.65%	11.49%	[59]
	C20:1 Eicosenoic acid	22–24 °C/24 h	1.83%	1.82%	[59]
	C22:1 cis Erucic acid	22–24 °C/24 h	1.86%	2.24%	[59]
	Saturated fatty acids	22–24 °C/24 h	10.58%	8.59%	[59]
	Polyunsaturated fatty acids	22–24 °C/24 h	61.53%	65.52%	[59]
	ω-6/ω-3		5.24	4.55	[59]
Barley	C16:0 Palmitic acid	20 °C/6 days	20.14 ± 0.150%	19.97 ± 0.388%	[57]
	C18:0 Stearic acid	20 °C/6 days	1.65 ± 0.379%	2.05 ± 0.086%	[57]
	C18:1 cis Oleic acid	20 °C/6 days	15.10 ± 0.380%	14.16 ± 0.415%	[57]
	C18:2 cis Linoleic acid (Omega-6)	20 °C/6 days	56.65 ± 0.316%	56.50 ± 0.804%	[57]
	C18:3n-3 Linolenic acid (Omega-3)	20 °C/6 days	4.52 ± 0.015%	6.73 ± 0.428%	[57]
	C20:1	20 °C/6 days	0.65 ± 0.303%	0.45 ± 0.041%	[57]
	ω-6/ω-3		12.53	8.39	[57]
Buckwheat	C16:0 Palmitic acid	37 °C/7 days	14.6%	15.8%	[72]
		25 °C/8 days	17.7 ± 1.9%	14.6 ± 0.8%	[73]
		30 °C/3 days	16.56 ± 0.03%	13.90 ± 0.01%	[58]
	C18:0 Stearic acid	37 °C/7 days	2.6%	6.7%	[72]
		25 °C/8 days	1.8 ± 0.1%	1.5 ± 0.2%	[73]
		30 °C/3 days	2.24 ± 0.02%	1.64 ± 0.02%	[58]
	C18:1 cis Oleic acid	37 °C/7 days	53.8%	61.4%	[72]
		25 °C/8 days	36.8 ± 2.1%	15.4 ± 1.4%	[73]
		30 °C/3 days	39.95 ± 0.1%	36.18 ± 0.09%	[58]
	C18:2 cis Linoleic acid (Omega-6)	37 °C/7 days	27.9%	14.9%	[72]
		25 °C/8 days	38.1 ± 2.5%	51.1 ± 3.0%	[73]
		30 °C/3 days	32.16 ± 0.11%	40.19 ± 0.15%	[58]
	C18:3n-3 Linolenic acid (Omega-3)	25 °C/8 days	2.7 ± 0.1%	18.9 ± 0.8%	[73]
		30 °C/3 days	1.64 ± 0.05%	2.84 ± 0.02%	[58]
	C20:0 Arachidic acid	25 °C/8 days	1.1 ± 0.3%	—	[73]
		30 °C/3 days	1.87 ± 0.01%	1.12 ± 0.01%	[58]
	C20:1 Eicosenoic acid	30 °C/3 days	4.18 ± 0.02%	3.10 ± 0.01%	[58]
	Saturated fatty acids	30 °C/3 days	21.2 ± 0.06%	16.84 ± 0%	[58]
	Monounsaturated fatty acids	30 °C/3 days	44.68 ± 0.16%	40.05 ± 0.03%	[58]
	Polyunsaturated fatty acids	30 °C/3 days	33.8 ± 0.16%	42.96 ± 0.08%	[58]
	ω-6/ω-3		-	-	[72]
	ω-6/ω-3		14.1	2.7	[73]
	ω-6/ω-3		19.6	14.15	[58]
Wheat	C11:0 Undecanoic acid	20 °C/3 days	1.7%	1.7%	[55]
	C16:0 Palmitic acid	30 °C/2 days	19.0%	19.0%	[61]
		17 °C/9 days	18.22 ± 0.46%	18.35 ± 2.10%	[74]
			18.76 ± 0.51%	18.77 ± 3.40%	
		20 °C/3 days	31.2%	33.5%	[55]
	C18:0 Stearic acid	30 °C/2 days	1.0%	1.1%	[61]
		17 °C/9 days	1.3 ± 0.35%	1.25 ± 0.15%	[74]
			2.44 ± 0.35%	4.13 ± 3.71%	
		20 °C/3 days	1.9%	1.2%	[55]
	C18:1 cis Oleic acid	30 °C/2 days	14.4%	14.1%	[61]
		17 °C/9 days	15.90 ± 0.23%	8.56 ± 0%	[74]
			18.56 ± 0.19%	14.35 ± 5.04%	
		20 °C/3 days	10.7%	7.8%	[55]
	C18:2 cis Linoleic acid (Omega-6)	30 °C/2 days	59.9%	59.9%	[61]
		17 °C/9 days	59.09 ± 1.67%	53.90 ± 3.40%	[74]
			52.22 ± 1.66%	47.44 ± 14.55%	
		20 °C/3 days	25.6%	27.3%	[55]
	C18:3n-3 Linolenic acid (Omega-3)	30 °C/2 days	4.0%	4.2%	[61]
		17 °C/9 days	4.04 ± 0.16%	16.08 ± 1.50%	[74]
			3.93 ± 0.12%	12.26 ± 1.36%	
		20 °C/3 days	2.0%	2.5%	[55]
	C22:0 Behenic acid	20 °C/3 days	1.2%	1.2%	[55]
	C20:3n-6 Eicosatrienoic acid	20 °C/3 days	1.4%	1.5%	[55]
	C20:3n-3 Eicosatrienoic acid	20 °C/3 days	2.3%	0.2%	[55]
	Saturated fatty acids	30 °C/2 days	20.3%	20.4%	[61]
	Polyunsaturated fatty acids	30 °C/2 days	64.0%	64.1%	[61]
	ω-6/ω-3		14	14	[61]
	ω-6/ω-3		14.62	3.35	[74]
	ω-6/ω-3		13.29	3.87	[74]
	ω-6/ω-3		12.8	10.92	[55]
Oat	C16:0 Palmitic acid	18 °C/4 days	16.53 ± 0.06%	16.64 ± 0.08%	[45]
	C18:0 Stearic acid	18 °C/4 days	34.19 ± 0.08%	33.09 ± 0.06%	[45]
	C18:1 cis Oleic acid	18 °C/4 days	34.19 ± 0.08%	33.09 ± 0.06%	[45]
	C18:2 cis Linoleic acid (Omega-6)	18 °C/4 days	43.73 ± 0.21%	44.36 ± 0.10%	[45]
	C18:3n-3 Linolenic acid (Omega-3)	18 °C/4 days	1.67 ± 0.01%	1.96 ± 0.01%	[45]
	Total saturated fatty acids	18 °C/4 days	18.19 ± 0.12%	18.30 ± 0.13%	[45]
	Total monounsaturated fatty acids	18 °C/4 days	36.29 ± 0.12%	35.26 ± 0.05%	[45]
	Total polyunsaturated fatty acids	18 °C/4 days	45.52 ± 0.22%	46.44 ± 0.10%	[45]
	ω-6/ω-3		26.2	22.6	[45]

## Data Availability

Data is contained within the article.
